# Investigating the effect of self-management program on stroke’s patients’ self-efficacy

**DOI:** 10.1186/s12883-022-02876-y

**Published:** 2022-09-22

**Authors:** Fereshteh Shalforoosh Amiri, Shahla Abolhassani, Nasrollah Alimohammadi, Tayebeh Roghani

**Affiliations:** 1 Faculty of Nursing and Midwifery, Isfahan University of Medical Sciences, Isfahan, Iran; 2 Adults Health Nursing Department, Faculty of Nursing and Midwifery, Nursing and Midwifery Care Research Center, Isfahan University of Medical Sciences, Hezar Jerib street, Isfahan, 81746-73461 Iran; 3 Department of Critical Care Nursing, School of Nursing and Midwifery, Isfahan University of Medical Sciences, Isfahan, Iran; 4 Department of Physical Therapy, Faculty of Rehabilitation Sciences, Isfahan University of Medical Sciences, Isfahan, Iran

**Keywords:** Stroke, Self-efficacy, Self-management, 5A self-management model

## Abstract

**Background and aim:**

Stroke patients face various challenges that affect their self-efficacy. The purpose of this study is to evaluate the effect of a self-management program on the self-efficacy of patients with Stroke.

**Methods and materials:**

This study is a clinical trial, in which 72 patients with stroke participated in this study. They were selected based on the convenience sampling method and assigned to either intervention or control group (36 patients in each group) randomly. The intervention group received 5A based self-management program for 6 weeks (in-person and off-site) and the control group received only routine care includes stroke training booklets and post-discharge care training by the ward nurse. Data were collected through demographic and jones self-efficacy questionnaires, before, immediately after, and 3 months after interventions in both groups and were analyzed with descriptive and analytical statistics using SPSS software (with independent t-test, Chi-square, Fisher and analysis of variance with repeated measures with a significance level of 0.05).

**Results:**

Before interventions, the two study groups had no statistically significant difference regarding demographic variables and the mean score of self-efficacy. Immediately and 3 months after interventions, the mean score and mean changes of self-efficacy score in the intervention group were significantly greater than in the control group(*p* < 0.001).

**Conclusion:**

The results of the present study show the appropriate effect of self-management program on self-efficacy of stroke patients. These results can be used by different members of healthcare teams to improve patients’ self-efficacy.

**Trial registration:**

This study is registered by Iranian Registry of Clinical Trials with decree code: IRCT20190712044181N2 (registration date: 05-11-2019).

## Introduction

Stroke is one of the leading causes of chronic disability in adults around the world. Stroke is a brain disorder of vascular origin, where its clinical manifestations rapidly progresses, either focal or diffuse, and lasts more than 24 hours. Twenty million people worldwide experience a stroke each year [1] and it is estimated that between 133 and 149 per 100,000 people experience a stroke in Iran annually [2].

Stroke can cause many complications [3]. For example, sudden changes in a person’s life after that can cause a variety of psychological and behavioral effects such as anxiety, despair, and other mood disorders. Another major complication experienced by patient is reduced mobility, which leads to inability to perform daily activities, job loss, and social isolation [4, 5]. Cognitive disorders are also among common complications of this disease that have a direct impact on quality of life and ability to perform daily functions [6]. Therefore, after discharge from hospital, patients will face new challenges. These have many consequences for them and adversely affects their sense of self-efficacy [7]. Self-efficacy is defined as a person’s self-confidence and belief in his or her ability to perform specific tasks [8–10]. According to Bandura’s social cognitive theory, self-efficacy greatly determines emotions, thoughts, motivation, and health-related behaviors. Self-efficacy also influences the goals set by individuals, the endeavor to achieve those goals, and the degree of resilience of individuals when faced with certain difficult situations [11]. high level sense of self-efficacy is essential for achieving maximum independence in performing basic daily tasks and is directly related to the quality of life in stroke patients [12]. Therefore, evaluating the level of self-efficacy and efforts to improve this feature in stroke patients can help them gain more control over many important aspects of their lives [13, 14].

Nowadays, there are various potential interventions to improve the self-efficacy of patients with chronic diseases, such as stroke, including self-management programs and behavior modification. Self-management programs can improve patients’ awareness and skills. It also promotes a health-oriented lifestyle [15]. Self-management is the process by which individuals learn the skills, strategies, and knowledge needed to manage the physical, psychological, emotional, and social symptoms caused by a chronic illness [16, 17]. Although the stroke-related care and self-management process begins at the time of hospitalization, the bulk of the process actually takes place after discharge and at home. This is achieved by using an appropriate self-management model, health-related decisions and care. It also requires participation of the patient, family members, and the treatment team. In addition, professional members of the treatment team, including nurses, can assist patients by using a coherent self-management intervention framework [13]. Self-management model 5A or “behavior change counseling” is one of the nursing-specific models and is an evidence-based approach that has been introduced with the aim of correcting patient’s behavior and achieving appropriate health. Introduced in 2002 by Glasgow et al., It includes five phases: Assess, Advise, Agree, Assist, and Arrange [18]. The role of the nurse in the this model is to perform self-management interventions when the patient is hospitalized, during consultations and outpatient visits, or during home care [19].

According to the results of studies on the implementation of self-management program in various diseases [16, 18, 20–25] and the lack of study on the impact of self-management program individually and based on Model 5A in patients with stroke in Iran (despite searching the multiple databases), and due to profound effects of stroke on all aspects of life, changing roles and numerous problems related to the failure to manage the critical conditions of patients and their family members, the central role that patients can play in managing their disease, the lack of providing adequate education and support to stroke patients and considering the role of the nurse in improving the performance of stroke patients and their family members, the present study was conducted to determine the effect of self-management program on self-efficacy of patients with stroke at the time of discharge from selected hospitals in province Isfahan, Iran.

## Materials and methods

This research was an interventional study (IRCT20190712044181N2,registration date: 05-11-2019), with intervention and control groups, which after obtaining the necessary permits from the ethics committee of Isfahan University of Medical Sciences (IR.MUI.RESEARCH.REC.1398.387) was performed in selected hospitals in Isfahan between October 2019 and September 2021 .

In this study, using the formula $n=\frac{{\left({z}_1+{z}_2\right)}^2{(2s)}^2}{d^2}$ at least 32 people were obtained and 36 people were selected in each group, taking into account 10% sample drop. Z1 at 95% confidence level was 1.96. Z2 of test power factor of 80% was equal to 0.84. S is an estimation of the average standard deviation of each of the variables in the two groups (experimental and control), and D is the minimum difference between the mean of each variable between the two groups, which shows a significant difference. S was considered 0.7.

Sampling was performed from the patient’s population ready to be discharged from the neurology sections hospitals of Isfahan-Iran. Patients were previously diagnosed with stroke (confirmed by a neurologist) and had study-specific inclusion criteria. The study was performed by continuous sampling method according to quota based on the number of beds in the relevant wards in each hospital, Random allocation of the studied units was done by selecting the card in such a way that the researcher had two cards that were placed in white and similar envelopes and on one of the cards the letter “I” (equivalent to the intervention group) and on the other the letter “C” (Equivalent to the control group) was registered in front of the patient and the patient, if able, or with him, drew one of the cards. If the I card was removed by the patient, she/he would start in the intervention group and the patient with C card would be placed in the control group. This continued until the number of people in each group was completed and were placed in one of these two groups according to the content on the card. This continued until the number of people in each group was completed. The hospital-specific number of patients were as follows: Kashani (20 patients), Amin (15 patients), Gharazi (17 patients) and Shariati (20 patients).

Inclusion criteria for patients included age (> 54 years) [26, 27],, lack of global and perceptual aphasia, ability to communicate (at least minimally), having a stroke for the first time, being in one of the four levels of Barthel dependence, not participating in other stroke-related training interventions and having the discharge order given by the treating physician.

Exclusion criteria included,, hospitalization due to a debilitating illness that interferes with the implementation of the program, communication barriers (cognitive disorder based on short form of mental state, mental disorder, blindness, deafness, mental retardation) or death during the study.

Furthermore, Inclusion criteria for family members included family members or relatives who could stay at home with the patient and are aware of the patient’s physical and mental conditions. This family member must also be involved in activities related to the patient, and be able to attend the several of this study from the beginning, follow-up visits to the end of the study stages.

The data collection tool in this study was a questionnaire. The first part of the questionnaire related to personal and clinical characteristics of the sample under investigation included questions related to age, sex, marital status, level of education, occupation, place of residence, health insurance coverage, history and type of underlying disease and type of stroke and muscle strength in four limbs as well as his Barthel’s score. Barthel score is included a list of daily life activities including eating, bathing, personal hygiene, dressing, defecating, how to use the toilet, stairs, getting out of bed and walking. It contains 11 items, which are scored differently depending on the ability level of the person in each of the five-option Likert scale for each question. In total, a person’s ability in different dimensions of daily activities is determined from zero to one hundred, and higher scores indicate a better situation, so that scores from 0 to 20 are completely dependent, 21 to 60 are severe dependence, 61 to 90 are moderate dependence, and 91 to 99 are partial dependence. And 100 is considered completely independent.

The second part of the questionnaire was related to stroke self-efficacy. The Jones Stroke Self-Efficacy Questionnaire, is a standard questionnaire that contains 13 questions related to stroke self-efficacy that are about the patient’s belief in doing things or how difficult they are after a stroke. Questions 1 to 8 are related to activity and questions 9 to 13 are related to self-efficacy. For each question, a line diagram is drawn, marked from 0 to 10 with a midpoint of 5, and they are asked to mark the number they feel confident they are able to perform at. The point zero indicates complete uncertainty and the point 10 indicates complete confidence. Options 1 to 5 indicate low confidence, while options 6 to 9 is considered medium confidence and option 10 is complete confidence [28]. This questionnaire was initially translated into Persian by the researcher to be used by the patients. It was subsequently translated back to English by a person fluent in English. Finally, it was thoroughly reviewed by two faculty members at the University of Isfahan and were edited and approved. To confirm the validity, the questionnaire was given to 16 faculty members, with using the Waltz content validity index calculation tool, a CVI index higher than 0.79 was measured for each item. The retest method was used to confirm the questionnaire. To do this, a questionnaire was given to 28 patients and was given to the same patients again within a certain period of time and the correlation coefficient between the two tests was determined by a statistical expert. The alpha was calculated as 0.89. it should be mentioned that the base case alpha scale is 0.90 [28], while the Chinese version is 0.92 [29], and in the Turkish version (Cronbach’s alpha), it varies between 0.92 and 0.93 [30].

The researcher made a needs assessment questionnaire in the form of a checklist of major problems in three areas of knowledge with three options (I do not have the necessary knowledge, I have some knowledge, but my knowledge is insufficient, I have the necessary knowledge), attitude with five options (completely agree), agree, have no opinion, disagree, completely disagree) and the performance is compiled with five options (always, often, sometimes, rarely and never) and is a tool for assessing the needs of stroke patients. Face validity and content validity were used to determine the validity of the questionnaire created by the researcher. The educational content was also prepared after studying the references and valid and approved articles by the relevant professors and the credibility was determined using content and face validity. In order to check the validity of the content and form of the questionnaire made by the researcher to determine the educational needs of the patients, we gave it to 16 people with opinions and after collecting their opinions and applying it, it was used. For the reliability of this questionnaire, the retest method was used. In order to retest, the questionnaire was given to 10 stroke patients who met the inclusion criteria, and after 2 weeks, it was given to the same patients again, and the correlation coefficient between the two tests was determined to be 0.75. CVR is a method of assessing the validity of a questionnaire, was designed by Lawshe. In order to calculate this ratio, the opinions of experts are used in the field of the desired test content. First, the objectives of the test are explained to the experts and the operational definitions related to the content of the questions are stated. They are then asked to rate each question on a three-point Likert scale:

- The subject is essential
- It is useful but not necessary
- It is not necessary

The content validity index or CVI was calculated in this questionnaire like the self-efficacy questionnaire. At the end, the patient and family members were asked to indicate if there was a need for further education and if there was any gap in the questionnaire. This questionnaire was given to the participants before the interventions. For sampling, the researcher referred to the neurology department of the study location, extracted the eligible items from the list of patients in the study community, and obtained informed consent from them to participate in the study. Participants were then randomly assigned to intervention and control groups. In the initial meeting with the participants with the inclusion criteria, the researcher placed two similar envelopes containing the cards marked with certain letters for the test and control group in front of the patient and the family. If capable, the patient (or his family) would withdraw a card and based on the card he would be placed either in control or test groups. Self-management program sessions started for the intervention group at the time of discharge and continued at home after the patient was discharged from the hospital. The study subjects were followed for up to 12 weeks After completing the intervention, Immediately after the intervention and 3 months after completing the intervention [31], the relevant questionnaire was completed again by the participants. In this study, the five stages of 5A self-management model including review, guidance, agreement, assistance and follow-up (during six intervention sessions and one follow-up session) were performed. Due to COVID disease, interventions were performed remotely. The first stage or the evaluation stage was performed on the individual patient, at the time of discharge. At this stage, the researcher, using a “needs assessment questionnaire”, examined the needs of participants in three areas of knowledge, attitude and practice. In the second stage or guidance, the researcher, according to the results obtained in the first stage, informed the research subjects and families about the health threats to patients (mentioned in the first stage) and the importance and benefits of behavioral change.

The first and second steps were performed in the first session and individually (The first two stages of the 5A model, that is, assess and advice, were implemented in the first session of our self-management program. This work was performed individually on each patient’s bed during discharge). In this meeting, the clients were handed over their self-report checklist (This checklist is to ensure the correct implementation of the program and to encourage the patient to follow the intervention). The third stage or agreement was done via phone and in the second session. At this stage, according to the identified problems (declared needs) in the first stage, an agreement was reached between the instructor and the learner on the development of behavioral goals appropriate to the found problem. Then, for each of the behavioral goals, a criterion was identified so that the participants could determine their level of confidence in the program implementation. Participants or the caregiver were asked, if possible, to record his or her performance status for each one of the behavioral goals in his or her reporting checklist on a daily basis for 3 months. In the fourth stage or cooperation, the trainings were presented individually according to the 5 top priorities set in the individual “needs assessment questionnaire”,In this way, after determining the level of physical, movement and psychological problems of each patient, the contents were changed according to the opinion of experts. At this stage, the educational content was developed based on behavioral goals and was approved by expert faculties in the same field: e.g., the type of physiotherapy exercises with the approval of a physiotherapist. The meetings were virtual (in the form of multimedia performances and film, animation and CD presentation). Questions and responses were collected at the appointed time via internet (Whatsapp messenger). Training included information on treatment conditions, medication management, symptom management (sleep management, relaxation, fatigue management), management of psychological components (anger management, coping with depression, adjustment, emotion control, and stress and anxiety management), lifestyle (exercise, nutrition and diet, smoking), social support (family support, communication with family members), effective communication (communication strategies) and problem-solving skills and decision-making skills. The assist phase started from the third session (third week) and continued until the sixth session (sixth week). At this stage, the training was done with the help of professors related to psychiatry and physiotherapy with the coordination and participation of the nurse. The fifth stage or follow-up was performed individually in the seventh session. This step was done via phone and in person. At this stage, the problems, questions and possible educational needs raised by the participants were also answered (Table 1). The trainings were determined based on the 5 basic needs determined by the patient and her/his family, and the content was provided to them based on their individual needs. The nurse’s role in the 5A self-management model is participation and cooperation with the patient and family members with the aim of facilitating the self-management process and motivating people to manage their illness every day. Nurses can provide these self-management interventions during the patient’s hospitalization, during consultations and outpatient visits, or during home care.

**Table 1:** Steps of self-management program

Session	Step	Goals	Content of the session	References	Presentation method	Trainer	Time
First	Assess and advise	Getting acquainted with the disease Recognize the dangers and benefits of behavior change	Completion of questionnaire, disease definition, etiology and prevention, prognosis, importance of treatment, care in relapse prevention with emphasis on threatening risks and benefits of behavior change	1-The Clincal Practice Of Neurological And Neurosurgical Nursing 8th edition (July 9, 2019) 2- Neuroscience Nursing, 2nd edition (January 1, 1900)	Multi media	Researcher	At least an hour
Second	Agree	Agree to set and accomplish behavioral goals	Agree on solving problems according to the patient’s priority, setting behavioral goals, introducing resources and services of the service provider		By phone	Researcher	
Third	Assist	Recognize the symptoms and deal with it correctly Familiarity with lifestyle changes, familiarity with medications, benefits and side effects	How to care for and deal with symptoms, familiarity with the importance and necessity of lifestyle changes (activity and exercise, diet and tobacco, drug management)	1- Fast Fact For Stroke Care Nursing,1st edition (May 7, 2014) 2- The Clincal Practice Of Neurological And Neurosurgical Nursing, Seventh edition (December 11, 2013)	Multimedia and Q&A in cyberspace and telephone	Researcher	Weekly
Fourth	Assist	Familiarity with anxiety and stress management methods and effective communication methods and familiarity with problem-solving and decision-making management methods and adaptation skills	Managing accompanying symptoms and providing effective communication methods, managing fatigue, coping with stress, and managing anxiety and depression, and developing coping skills, problem-solving methods, and decision-making.	1-Psychiatric Mental Health Nursing, Ninth edition,2018 2 -Coping With Life Challenges, 2nd edition (February 1, 2002) 3- Complete collection of life skills, 10th edition, 2013	Multimedia (tailored to the priorities in the needs assessment questionnaire)	Researcher And Psychiatric nurse	
Fifth	Assist	Preparing to start moving and how to do it, appropriate movement exercises	Condition management and rehabilitation (stages, duration, amount by providing educational booklets and videos of educational cases	1-Adult Hemiplegia Evaluation & Treatment Bobath,3th edition,june 1990 2-Physical Rehabilitation (Stroke), 1st edition (May 23, 2012)	Multimedia Screening videos (according to the facilities of patients’ homes) and questions and answers in cyberspace or by phone	Researcher And Physiotherapist	

The control group, during the intervention and 3 months after the end of the follow-up, were contacted by phone once a month at the same time, and their conditions were evaluated by the personnel in order to perform the routine care during discharge. They were followed up and asked to perform these routines. Also, to comply with the ethics, after measuring the variables in the post-test stage, the educational content provided to the intervention group was given to the control group.

Data obtained in the present study was processed using descriptive statistics (frequency, mean, standard deviation) and inferential statistics (Fisher’s exact test, chi-square test, paired t-test and analysis of variance with duplicate values). SPSS software Version 22 was used for analysis. In all statistical tests, a *p*-value of less than 0.05 was considered.

## Results

The mean age of participants in the intervention group was 69.14 (8.64) and in the control group was 67.7(8.30). Also, 38.9% of the participants in the intervention group and 52.8% of the participants in the control group were female. The results obtained in the present study showed that the two groups did not show a statistically significant difference in terms of demographic variables (*P* > 0.05). The demographic variables of the participants are shown in Tables 2, 3, 4.

**Table 2:** Comparison of mean age, mental status score and Barthel score in intervention and control groups

Variable	Group	Number of participants	Mean ± standard deviation	Independent T test results	
				T	P
Age	Intervention	36	69.1(8.64)	0.737	0.464
	Control	36	67.67(8.30)		
Barthel score	Intervention	36	58.47(12.24)	1.05	0.298
	Control	36	55.50(11.78)		
Mental state score	Intervention	36	23.89(1.21)	0.698	0.487
	Control	36	23.67(1.47)

**Table 3:** Comparison of frequency distribution of education, occupation, place of residence, type of stroke, marital status and organ with reduced muscle strength in the intervention and control groups

Variable		Intervention group		Control group		Fisher precise test	*P* value
		Number	Percent	Number	Percent		
Education level	Illiterate	5	13.9%	1	2.8%	3.568	0.482
	Elementary	9	25%	9	25%		
	Elementary School	8	22.2%	12	33.3%		
	High School	11	30.6%	10	27.8%		
	College	3	8.3%	4	11.1%		
Occupation	Employed	0	0	0	0	7.059	0.113
	Self-employed	1	2.8%	7	19.4%		
	House Keeper	12	33.3%	12	33.3%		
	Unemployed	0	0%	0	0%		
	Worker	2	5.6%	0	0%		
	Retired	15	41.7%	14	39.9%		
	Others	6	16.7%	3	8.3%		
place of Residence	With their Children	6	16.7%	3	8.3%	2.009	0.588
	Own Home	22	61.1%	27	75%		
	Renter	6	16.7%	4	11.1%		
	Others	2	2.8%	2	2.8%		
Type of Brain Stroke	Ischemic	35	97.2%	36	100	0.00	1
	Hemorrhagic	1	2.8%	0	0		
Marriage Status	Single	2	5.6%	3	8.3%	0.619	1
	Married	26	72.2%	26	72.2%		
	Widowed	7	19.4%	6	16.7%		
	Divorced	1	2.8%	1	2.8%		
The Muscle Involved	Right Hand	4	11.1%	5	13.9%	5.075	0.260
	Left Hand	0	0	3	8.3%		
	Right Leg	0	0	0	0		
	Left Leg	0	0	0	0		
	right Hand and Leg	13	36.1%	15	41.7%		
	Left Hand and Leg	17	47.2%	13	36.1%		
	All four	2	5.6%	0	0

**Table 4:** Comparison of frequency distribution of gender, type of insurance, history of underlying disease, living alone, level of dependence, paralysis and number of organs with palsy between the intervention and control groups

variable		Intervention group		Control group		Chi-square test	*P* value
		Number	Percent	Number	Percent		
Gender	Female	14	38.9%	19	52.8%	1.39	0.344
	Male	22	61.1%	17	47.2%		
Insurance type	Uninsured	0	0	0	0%	0.363	0.834
	Social Security	23	63.9%	22	61.1%		
	Medical services	7	19.4%	9	25%		
	Armed Forces	0	0	0	0		
	Other	6	16.7%	5	13.9%		
	Complementary insurance	0	0	0	0		
	Free (self-processing)	0	0	0	0		
History of underlying disease	Yes	23	63.9%	23	58.3%	0.234	0.629
	No	13	46.4%	15	53.6%		
Living Alone	No	7	19.4%	7	19.4%	0.00	1
	Yes	29	80.6%	29	80.6%		
Dependency level based on Barthel scale	Full dependence	0	0	0	0	0.067	0.795
	Severe dependence	25	69.4%	26	72.2%		
	Average dependence	11	30.6%	10	27.8%		
	Partial dependence	0	0	0	0%		
	Independent	0	0	0	0%		
Paralysis of the limbs	Yes	27	75%	29	80.6%	0.321	0.571
	No	9	25%	7	19.4%		
Number of limbs with palsy (paralysis)	one limb	13	36.1%	12	33.3%	0.616	0.828
	Two limbs	14	38.9%	17	47.2%		
	No paralysis	9	25%	7	19.4%		

The results obtained in the present study showed that the two groups did not have a statistically significant difference in terms of mean self-efficacy score in the pre-intervention stage (*P* = 0.297). Also, the results of independent t-test showed that the two groups had a statistically significant difference in terms of mean self-efficacy score in the immediate aftermath (*P* = 0.047) and 3 months after the intervention (*P* = 0.009). The results of independent t-test also showed that the two groups had a statistically significant difference in terms of mean changes in self-efficacy score immediately after the intervention (*P* < 0.001). At the three-month point after the interventions, the mean change in self-efficacy score in the intervention group was 4.55 (3.42, 5.57) and in the control group was −0.41(−1.25, 0.43). The results of paired t-test showed that the two groups had a statistically significant difference in terms of mean changes in self-efficacy score in the 3 months after the intervention (Table 5). To compare the mean score of self-efficacy between the three time points (before, immediately after, a while after the intervention) in each of the test and control groups, the results were analyzed using analysis of variance with repeated measurements. According to the results of Mochelli test, the sphericity assumption was not established in the intervention and control groups (p in the intervention group = 0.002 and p in the control group was 0.00). Therefore, Greenhouse-Geisser test results were used to interpret the results and evaluate the significance of changes in self-efficacy over time (during implementation of interventions). The results of this test showed that over time (after the intervention and two post-test stages), the self-efficacy score in the intervention group increased significantly (*P* < 0.001), while in the control group, it did not change significantly over time (*P* = 0.244), The effect size is 0.582 in the test group and 0.039 in the control group (Table 5).

**Table 5:** Comparison of mean score and mean changes in self-efficacy score between intervention and control groups

Stage	Intervention group	Control group	Effect Size	*P* value
	Mean (%95 CI)	Mean (%95CI)		
Before the intervention	84.47(80.39, 88.54)	81.13(76.47, 85.79)	0.125	0.297
Immediately after the intervention	86.88(82.91, 90.85)	80.58(75.94, 85.22)	0.234	0.047
Three months after the implementation of the interventions	89.02(85.16, 92.88)	80.72(76.08, 85.36)	0.306	0.009
Mean changes immediately after interventions	2.41(1.51, 3.31)	−0.55(−1.27, 0.17)	0.513	< 0.001
Mean changes three months after the interventions	4.55(3.42, 5.57)	−0.41(−1.25, 0.43)	0.643	< 0.001

## Discussion

The aim of this study was to investigate the effect of self-management program on self-efficacy of stroke patients during discharge from selected hospitals in province of Isfahan, Iran. According to the interpretation of Jones self-efficacy questionnaire scores, the self-efficacy of the participants in the present study was at a moderate level in the pre-intervention stage and the two control and test groups did not differ in a statistically significant way in terms of mean pre-intervention self-efficacy score.

This result is consistent with the findings of Tielemans et al. [32]. On 110 patients with stroke in the where participants’ self-efficacy was also moderate. In the study of Long et al. [3]., The aim was to investigate the effect of training using virtual reality technology on self-efficacy of stroke patients in China. The mean self-efficacy score of the participants was higher than the average self-efficacy score of the participants in the present study. In a study by Sabariego et al. [11]. Conducted in Germany to investigate the effect of educational intervention on self-efficacy in stroke patients, the self-efficacy of participants in the pre-intervention stage was moderate, which is consistent with the results of the present study.

The results of the present study showed that the two groups had a statistically significant difference in terms of mean self-efficacy score immediately after and 3 months after completing the intervention and the mean self-efficacy score in the intervention group was significantly higher than the control group. Also, the mean changes of self-efficacy score in the stage immediately after and 3 months after completing the intervention in the intervention group were significantly higher than the control group.

Due to the fact that the two groups were not statistically significant in terms of contextual variables and the mean score of self-efficacy in the pre-intervention stage, it can be stated with 95% confidence that the higher mean score and mean changes in self-efficacy score after the interventions in the intervention group compared to the control group, was due to the interventions performed in the intervention group.

The results of study by Mirzaei et al.(2017) [33]., with the investigation of effect of self-management training program on self-efficacy of the elderly with knee osteoarthritis and the study.,this study by Kaveh Savadkooh et al.(2012) [21] With examining the impact of the self-management program on self-efficacy of patients with primary hypertension, consistent with the results of the present study in terms of the effectiveness of self-management intervention on self-efficacy.

The results of Heydari’s et al.(2016) study [20] with studying the effect of model 5A self-management program on the self-efficacy of patients with chronic obstructive pulmonary disease, and Golafshani et al. (2020) [23] with examining the effect of self-management program based on Model 5A on self-efficacy of the elderly with diabetes, consistent with the results of the present study in terms of the effectiveness of self-management intervention on self-efficacy. In these studies, the theoretical model used was similar to the present study.

In a 2018 study by Chen et al. [34]., The effect of a self-management program on self-efficacy and daily life activities in stroke patients was investigated. In this study, conducted in China, 144 patients with the first stroke were randomly assigned to intervention and control groups. The intervention group received a self-management program and underwent phone follow-ups for 4 weeks. The results of this study showed that self-management intervention was effective in promoting self-efficacy in stroke patients. These results are consistent with the results of the present study, however, the self-management intervention designed differs from the self-management intervention performed in the present study in terms of the number of sessions, how it is done (in-person and virtual) and follow-up time. In 2018, Lo et al. [25]. conducted a study to investigate the effect of self-management program on self-efficacy in stroke patients. In this study, conducted in Hong Kong, 128 patients with stroke were randomly assigned to intervention and control groups. The intervention group received a self-management program for 4 weeks. For the control group, only routine care was performed. The results of this study showed that the self-management program was effective in improving the level of self-efficacy of stroke patients. These results are consistent with the results obtained in the present study. In a 2018 clinical trial study, Damoush et al. [35] examined the effect of a self-management program on the self-efficacy of stroke patients. There were 258 participants in this study who were randomly assigned to intervention and control groups. A self-management program was implemented for the intervention group and the control group only received routine care. The results of this study showed that the intervention was effective in improving the self-efficacy of stroke patients 6 months after the intervention. These results are consistent with the results obtained in the present study.

In general, the results obtained in the present study as well as the above-mentioned studies show that the use of self-management can be useful in improving the self-efficacy of stroke patients.

## Conclusion

The results of the present study indicated that implementing self-management program on patients experience a stroke could improve self-efficacy among these patients. The results of this study can be used in patients at the time of discharge to increase self-efficacy, health service delivery systems, and lead to an increase in self-efficacy.

### Limitations

One of the limitations of this study was not examining the effects of implementing a self-management program for a period longer than 3 months. Another limitation of this study was the impossibility of attending patients’ homes due to the prevalence of Covid 19 disease and the small sample size.

## Data Availability

The datasets generated and analyzed during the current study are not publicly available due to the identity information contained in the data but are available from the corresponding author on reasonable request by deleting this information.
